# Joint effect of abnormal systemic immune-inflammation index (SII) levels and diabetes on cognitive function and survival rate: A population-based study from the NHANES 2011–2014

**DOI:** 10.1371/journal.pone.0301300

**Published:** 2024-05-06

**Authors:** Wanying Chen, Xinyue Sun, Jiaxin Han, Xiaoyu Wu, Qingfan Wang, Mengmeng Li, Xiangyu Lei, Yixuan Wu, Zhiheng Li, Guogang Luo, Meng Wei

**Affiliations:** Department of Neurology, The First Affiliated Hospital of Xi ’an Jiaotong University, Xi’an, China; Hamadan University of Medical Sciences, School of Public Health, ISLAMIC REPUBLIC OF IRAN

## Abstract

**Objective:**

The purpose of this study was to investigate whether the combination of abnormal systemic immune-inflammation index (SII) levels and hyperglycemia increased the risk of cognitive function decline and reduced survival rate in the United States.

**Methods:**

This cross-sectional study used data from the National Health and Nutrition Examination Survey (NHANES) database from 2011–2014 and enrolled 1,447 participants aged 60 years or older. Restricted cubic splines (RCS), linear regression and kaplan-meier(KM) curve were employed to explore the combined effects of abnormal SII and hyperglycemia on cognitive function and survival rate, and subgroup analysis was also conducted.

**Results:**

The RCS analysis revealed an inverted U-shaped relationship between lgSII levels and cognitive function. Linear regression analysis indicated that neither abnormal SII nor diabetes alone significantly contributed to the decline in cognitive function compared to participants with normal SII levels and blood glucose. However, when abnormal SII coexisted with diabetes (but not prediabetes), it resulted to a significant decline in cognitive function. After adjusting for various confounding factors, these results remained significant in Delayed Word Recall (β:-0.76, P<0.05) and Digit Symbol Substitution tests (β:-5.02, P<0.05). Nevertheless, these results showed marginal significance in Total Word Recall test as well as Animal Fluency test. Among all subgroup analyses performed, participants with both abnormal SII levels and diabetes exhibited the greatest decline in cognitive function compared to those with only diabetes. Furthermore, KM curve demonstrated that the combination of abnormal SII levels and diabetes decreased survival rate among participants.

**Conclusion:**

The findings suggest that the impact of diabetes on cognitive function/survival rate is correlated with SII levels, indicating that their combination enhances predictive power.

## 1. Introduction

The world’s population is aging rapidly, with virtually every country in the world reporting large increases in the number and proportion of older people [1]. This means that age-related cognitive decline has become an important social problem that concerns all of humanity and needs to be addressed urgently. The number of people with cognitive decline is increasing worldwide [2]. For example, in the United States, more than 6 million people over the age of 65 had been diagnosed with cognitive dysfunction by 2020, and that number is expected to increase to 13.85 million by 2060 [3]. The increase of cognitive impairment will lead to an increase in the incidence of various geriatric diseases, which will increase the investment of medical resources and cause a heavy socioeconomic burden [4,5]. Therefore, more effective prevention and control measures are urgently needed.

Cognitive decline is associated with chronic inflammation in the body, and older adults with high levels of inflammatory markers, including white blood cells, neutrophil-lymphocyte ratio (NLR), and neutrophil-albumin ratio (NAR), are at higher risk for cognitive impairment [6–8]. As simple markers of inflammation, their ability to predict cognitive impairment has been evaluated. But these markers involve only one or two types of immune inflammatory cells and may not accurately reflect the state of inflammation in the body. The systemic immune-inflammation index (SII) is a comprehensive novel inflammatory biomarker based on neutrophil, lymphocyte, and platelet counts that can represent different inflammatory and immune pathways in the body with greater stability [9]. SII is calculated as platelet count x neutrophil count/lymphocyte count [10]. It is now thought to accurately reflect inflammation [11]. Studies have shown that older adults with higher levels of dietary inflammation and SII are at higher risk for cognitive impairment [6]. Diabetes, a recognized risk factor for cognitive impairment, may be primarily due to vascular complications associated with abnormal blood sugar, including brain small vessel lesions and stroke [12,13]. Epidemiological review studies have proved that cognitive dysfunction is a complication of diabetes [14,15] and even prediabetes [16]. The Framingham Heart Study demonstrated that hyperglycemia is related to impaired attention and memory in people with undiagnosed diabetes and prediabetes [17]. Remarkably, there is a association between inflammatory biomarkers and the development of diabetes and its complications [18]. The study showed that older participants with lower cognitive functions have an increased risk for all-cause and cardiovascular disease mortality compared to older participants with a higher level of cognitive function [19].

Our hypothesis is that the co-occurrence of hyperglycemia and abnormal SII levels increases the risk of cognitive decline and decreases survival rate.

Therefore, we aimed to assess the combined effect of hyperglycemia and abnormal SII on cognitive function and survival rate in older adults using data from the National Health and Nutrition Examination Survey (NHANES) database from 2011–2014

## 2. Material methods

### 2.1 Study population

We obtained data from the NHANES database, which is conducted by the Centers for Disease Control and Prevention’s National Center for Health Statistics (NCHS) to assess the health and nutrition of the population of the United States.

All data analyzed in the present study are freely available online at the CDC website.

https://www.cdc.gov/nchs/nhanes/index.htm

The data survey was a combination of interview and physical examination. The survey included demographic, dietary, socioeconomic, and health-related questions. In addition, the examination includes medical, dental and physiological assessments, as well as laboratory analyses performed by highly skilled medical practitioners. This data is based on stratified, multi-stage probability sampling and is representative of the US population. NHANES research had been approved by the NCHS Research Ethics Review Committee. All participants in the survey signed informed consent forms. No ethical or administrative permission is required to access the NHANES database.

**Fig 1:**
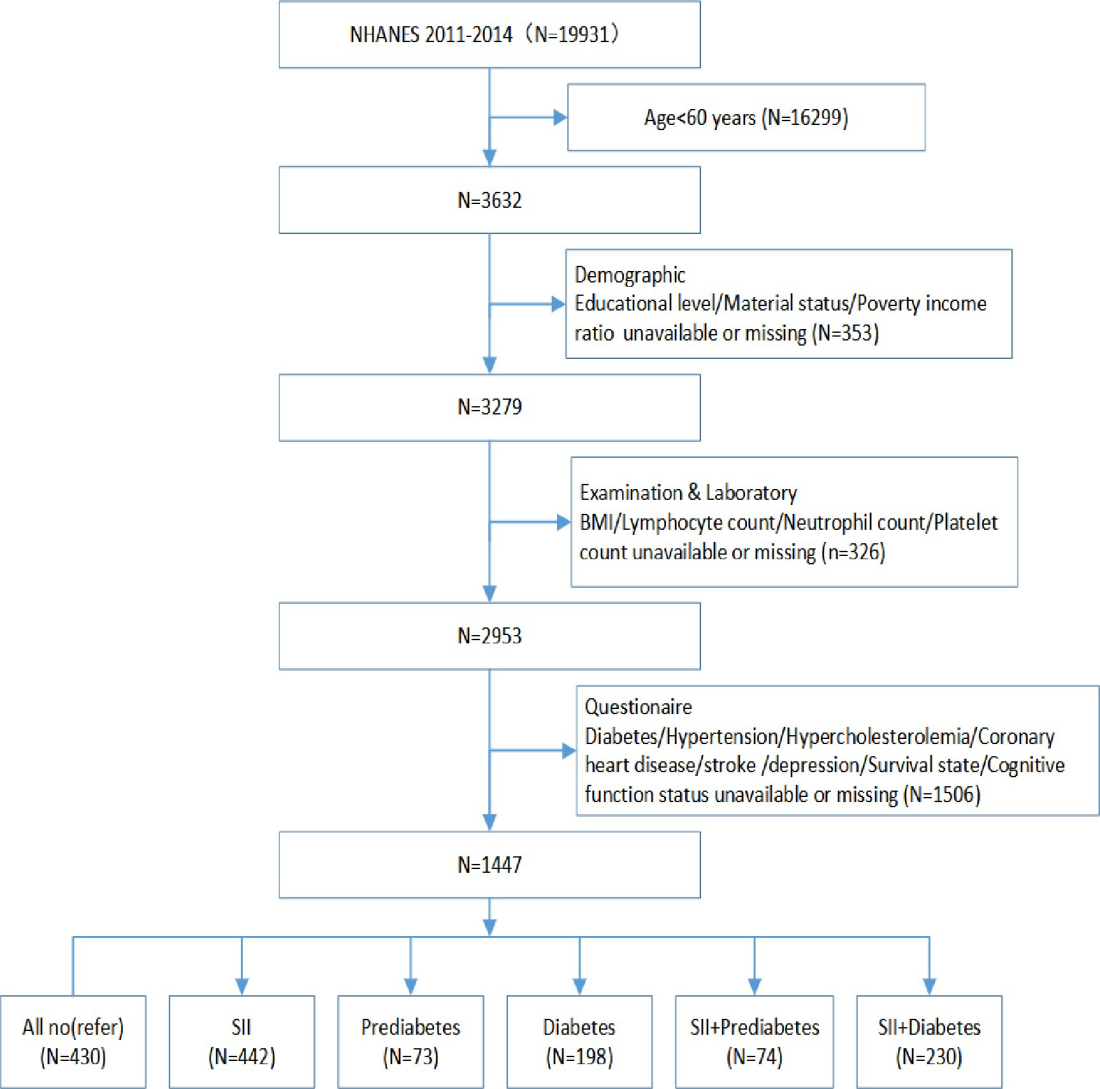
Flow chart of the study population inclusion. body mass index BMI.

This analysis uses data from two survey cycles in the NHANES database (2011–2012, 2012–2014). Individuals younger than 60 years of age or missing values for key analytical variables were excluded. The inclusion criteria for this study are summarized in the flow chart shown in Fig 1

### 2.2 Exposure and outcome definitions

Cognitive function assessment consisted of the following four tests [5]: Firstly, the Consortium to Establish a Registry for Alzheimer’s Disease Word Learning (CERAD-WL) test consisted of three consecutive learning experiments, which asked participants to immediately recall as many words as possible after reading 10 unrelated words aloud, with a total score of 30; Secondly, the CERAD Delayed Recall (CERAD-DR) test, which took place 10 minutes after the three continuous learning experiment, and participants were asked to recall words on the CERAD-WL test, is performed on a 10-point scale; The third is the animal fluency test, which assesses verbal fluency [20]. Participants were asked to name as many animals as they could within one minute, with each name receiving a share; Finally, the Digit Symbol Substitution Test (DSST) is a global measure of brain health, which measures processing speed, sustained attention, visual scanning and short-term memory [21]. The test required participants to draw the corresponding into a box next to a number within two minutes, totaling 133 boxes.

Diabetes is defined as follows: 1. diagnosed by a physician or health care professional; 2. HbA1c≥6.5%; 3.Fasting blood glucose> = 7.0mmol/L; 4. Random blood glucose> = 11.1mmol/L; 5. Two-hour OGTT blood glucose> = 11.1mmol/L; 6. Use of diabetes medication or insulin. Prediabetes is defined as follows: 1. Impaired fasting glycaemia: 6.11mmol/L< = fast glucose<7.0mmol/L; 2.Impaired glucose tolerance: 7.7mmol/L< = Two-hour OGTT blood glucose<11.1mmol/L. Diabetes and prediabetes are defined as hyperglycemia.

Blood specimens were measured at the NHANES Mobile Examination Centers (MECs). In 2011–2012 the Beckman Coulter MAXM was the hematology analyzer but in 2013–2014 the hematology analyzer used was the Beckman Coulter DXH 800. SII = platelet count x neutrophil count/lymphocyte count.

### 2.3 Variables

According to the available literature and clinical experience, variables with potential links to cognitive function decline were collected in this study. Demographic characteristics include age and sex (male/female), race (Mexican Americans, non-Hispanic Black, non-Hispanic white, other races), marital status (We put married/living with a partner in one category, widowed/divorced/separated in another, and never married in another), education levels (less than high school, high school, above high school) and poverty income ratio (divide into ≤1.2 and >1.2). In addition, examination include BMI. Laboratory test indicators include lymphocyte, neutrophil and platelet. Moreover, questionaire include the history of diabetes, hypertension, hypercholesterolemia, coronary heart disease, smoking and drinking, as well as daily energy intake, physical activity and depressive state. In addition, we also collected relevant survival data.

### 2.4 Statistical analysis

In this study, appropriate weights of NHANES samples were used for statistical analysis. Using RCS curve, the linear and nonlinear relationship between SII and cognitive function was studied after excluding confounding factors (age, sex, race, education level, marital status, poverty income ratio, smoking status, drinking status, hypercholesterolemia, hypertension, coronary heart disease, stroke, depressive state, Intake of energy and physical activity). However, SII was log-transformed when conducting RCS analysis, considering that these inflammatory markers were right-skewed distributed.Then SII was divided into 4 groups(Q1-Q4) according to the quartile, with Q1+Q4 as the abnormal group and Q2+Q3 as the normal group.

Continuous variables are expressed as the median (lower quartile-high quartile) and categorical variables are expressed as frequencies (percentages). For group comparisons of data with a normal distribution, we utilize weighted Student’s t-test or one-way ANOVA. For group comparisons of skewed variables, we used the weighted Wilcoxon test or Kruskal Wallis test. The weighted chi-square test was performed to analyze inter-group differences for categorical variables. We divided the participants into the following groups according to SII levels and blood glucose status: All no group, SII group, Prediabetes group, Diabetes group, SII+Predabetes, SII+Diabetes group (See Table 1 for detailed explanations), and then used weighted multiple linear regression analysis to explore the relationship between SII and blood glucose with cognitive function.

**Table 1:** Weighted baseline characteristics of participants in six groups: NHANES 2011–2014.

Variable	All no^a^	SII^b^	Prediabetes^c^	Diabetes^d^	SII+Prediabetes^e^	SII+Diabetes^f^	*P*
**Age(years)**	66.60(63, 72)	67(63, 73)	68(64, 76)	68(64, 74)	68(63, 77)	68(63, 76)	*0*.*06*
BMI (kg/m^2^)	27.4(24.2, 30.2)	26(23.9, 29.7)	28.4(24.8, 31.5)	29.2(26.5, 33.7)	**29.8(24.7, 33.4)**	30.6(26.4, 34.9)	<0.0001
**energy_kcal**	1871(15555.5, 2330.5)	1858.5(1506.5, 2353.5)	1996.5(1681.5, 2585)	1755.5(1367.5, 2221.5)	**1893(1508.5, 2386.5)**	1631(1249, 2029.5)	<0.001
**PA_total_MET(min/week)**	1440(600, 3420)	1760(840, 4140)	2160(960, 5040)	1200(680, 2880)	1440(480, 3360)	1120(480, 2400)	0.04
Sex, n (%) 0.29							
Female	236(56.25)	207(50.92)	37(41.64)	89(45.37)	29(45.85)	108(56.13)	
Male	194(43.75)	235(49.08)	36(58.36)	109(54.63)	45(54.15)	122(43.87)	
Race, n (%) <0.0001							
Mexican American	34(2.41)	20(1.33)	7(3.01)	20(4.44)	13 (5.32)	25(5.68)	
Non-Hispanic Black	70 (4.65)	109 (7.22)	8 (2.75)	43 (8.46)	14 (5.34)	70(13.25)	
Non-Hispanic White	237(85.41)	245(85.80)	45(88.92)	82(73.06)	44(86.09)	96(72.75)	
other	89 (7.53)	68 (5.65)	13 (5.32)	53(14.04)	9 (5.76)	39 (8.33)	
Educational level, n (%) <0.001							
<high	71 (8.51)	67 (9.12)	18(16.71)	46(15.19)	11 (9.01)	63(20.08)	
>high	271(72.93)	275(72.19)	45(76.44)	103(60.53)	45(71.98)	108(50.75)	
high	88(18.56)	100(18.69)	10 (6.85)	49(24.27)	18(19.01)	59(29.18)	
**Marital status,** n (%) **0.33**							
Married/Living with partner	273(70.53)	252(65.86)	44(65.86)	137(71.38)	51(72.42)	134(61.73)	
Never married	17(2.85)	30(4.57)	1(2.05)	7(1.58)	5(6.86)	16(6.50)	
Widowed/Divorced/Separated	140(26.62)	160(29.58)	28(32.09)	54(27.04)	18(20.73)	80(31.77)	
**Poverty income ratio,** n (%) **<0.001**							
≤1.2	76 (8.18)	78 (8.42)	12 (6.23)	41(13.33)	16(10.27)	67(20.33)	
>1.2	354(91.82)	364(91.58)	61(93.77)	157(86.67)	58(89.73)	163(79.67)	
**Alcohol.user,** n (%) **0.01**							
Former	94(17.04)	109(19.96)	16(14.57)	53(22.88)	17(15.71)	94(35.61)	
Heavy	27 (4.23)	26 (5.50)	10(10.50)	19 (9.38)	4 (7.21)	11 (4.78)	
Mild	204(52.18)	194(48.78)	30(50.32)	77(46.30)	37(59.38)	81(43.08)	
Moderate	58(18.04)	52(14.13)	7 (9.78)	13 (5.28)	6(11.41)	8 (2.95)	
Never	47 (8.51)	61(11.64)	10(14.84)	36(16.16)	10 (6.29)	36(13.58)	
**Smoke,** n (%) **0.19**							
Former	154(38.72)	171(39.80)	24(33.99)	87(51.58)	37(54.36)	95(42.73)	
Never	232(53.05)	219(49.58)	41(59.01)	90(42.31)	32(39.07)	110(46.55)	
Now	44 (8.23)	52(10.62)	8 (7.00)	21 (6.11)	5 (6.57)	25(10.72)	
**Hypertension,** n (%) **<0.0001**							
No	213(54.80)	210(53.04)	26(34.04)	63(28.86)	26(40.39)	62(26.10)	
Yes	217(45.20)	232(46.96)	47(65.96)	135(71.14)	48(59.61)	168(73.90)	
Hypercholesterolemia, n (%) <0.001							
No	204(44.78)	213(50.48)	28(37.80)	57(25.10)	35(39.63)	86(35.11)	
Yes	226(55.22)	229(49.52)	45(62.20)	141(74.90)	39(60.37)	144(64.89)	
Coronary heart disease, n (%) <0.001							
No	407(95.72)	409(90.94)	68(94.24)	171(87.35)	63(80.25)	197(84.18)	
Yes	23 (4.28)	33 (9.06)	5 (5.76)	27(12.65)	11(19.75)	33(15.82)	
**Stroke,** n (%) **0.24**							
No	415(97.14)	415(95.50)	72(98.58)	187(93.67)	**67(90.87)**	214(94.71)	
Yes	15(2.86)	27(4.50)	1(1.42)	11(6.33)	**7(9.13)**	16(5.29)	
**Depression,** n (%) 0.05							
No	411(95.53)	417(96.01)	69(96.00)	176(91.69)	69(96.73)	200(88.99)	
Yes	19 (4.47)	25 (3.99)	4 (4.00)	22 (8.31)	5 (3.27)	30(11.01)	

^a^AII no is normal SII, non-diabetes and non-prediabetes.

^b^SII is abnormal SII, non-diabetes and non-prediabetes.

^c^Prediabetes is prediabetes and normal SII.

^d^Diabetes is diabetes and normal SII.

^e^SII+Prediabetes is abnormal SII and prediabetes.

^f^SII+Diabetes is abnormal SII and diabetes.

For continuous variables: Median (lower quartile-high quartile), P-value was by weighted one-way ANOVA or Kruskal Wallis test. For categorical variables: Frequencies (percentages), P-value was by weighted Chi-square test. PA_total_MET: Physical activity_total_metabolic equivalent.

Model 1 was adjusted for gender, age, race, education, marital status, and income. Model 2 added BMI, smoking, alcohol consumption, hyperlipidemia, hypertension, coronary heart disease and stroke to Model 1. Model 3 was adjusted for all covariables. Kaplan–Meier curves and the log-rank test were used to compare the cumulative risk of events according to abnormal SII levels and diabetes. The subgroup analyses were performed to evaluate the combined effect of abnormal SII levels and diabetes on cognitive function, considering age (≤ 70 or > 70/years), sex and BMI (< 25 or ≥ 25 kg/m2).

Data analyses were completed using R software version 4.2.0 The RCS was created with the “rms” package.

## 3. Results

### 3.1 Relationship between SII and cognitive function

**Fig 2:**
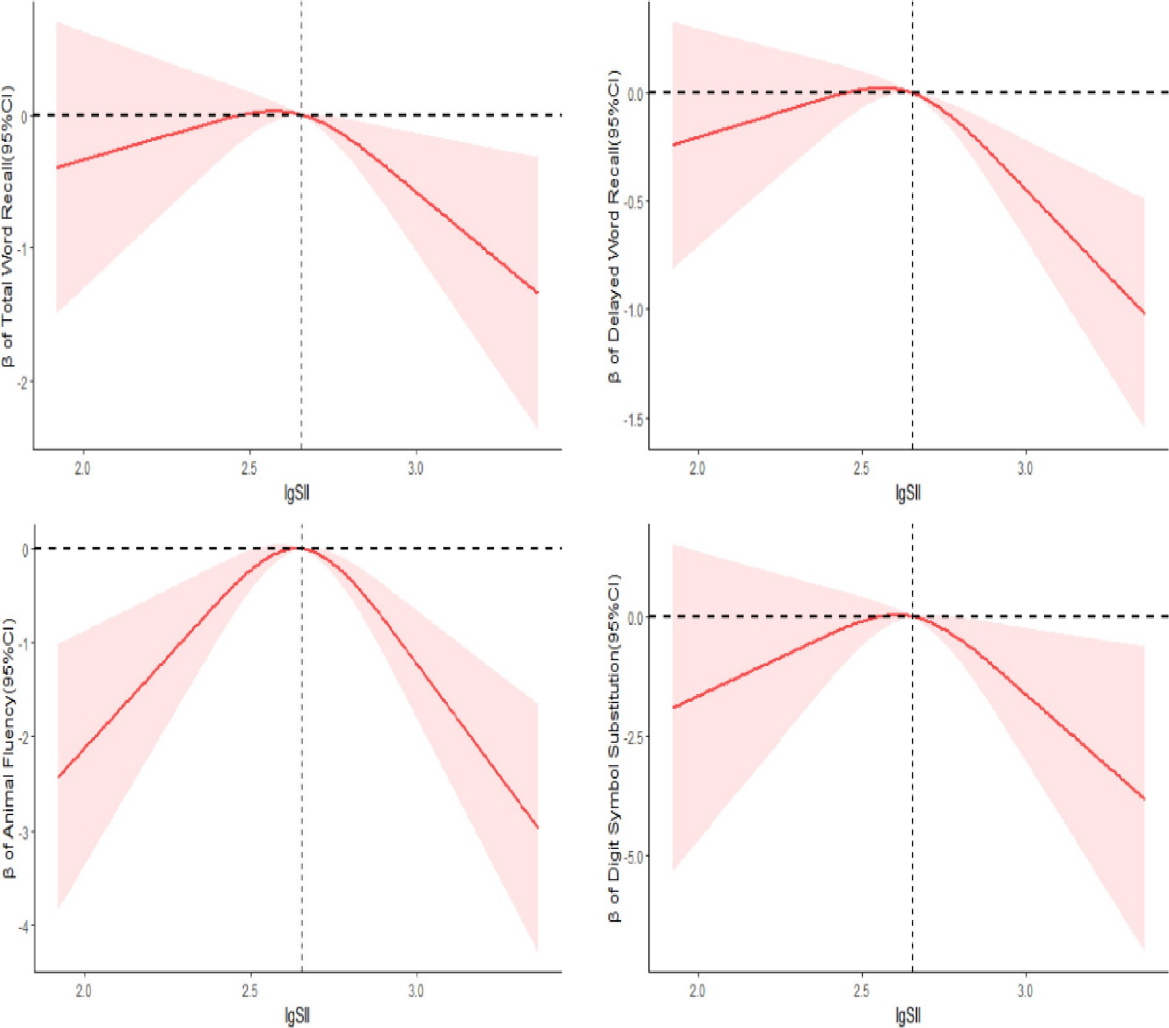
RCS was used to analyze the association between lgSII and cognitive function scores.The analysis was performed after adjusting for age, sex, race, education level, Marital status, Poverty income ratio, smoking status, drinking status, hypercholesteremia, hypertension, Coronary heart disease, stroke, depressive state, Intake of energy and Physical activity. The solid line represents β, and the shaded red represents 95% CI.

**Fig 3:**
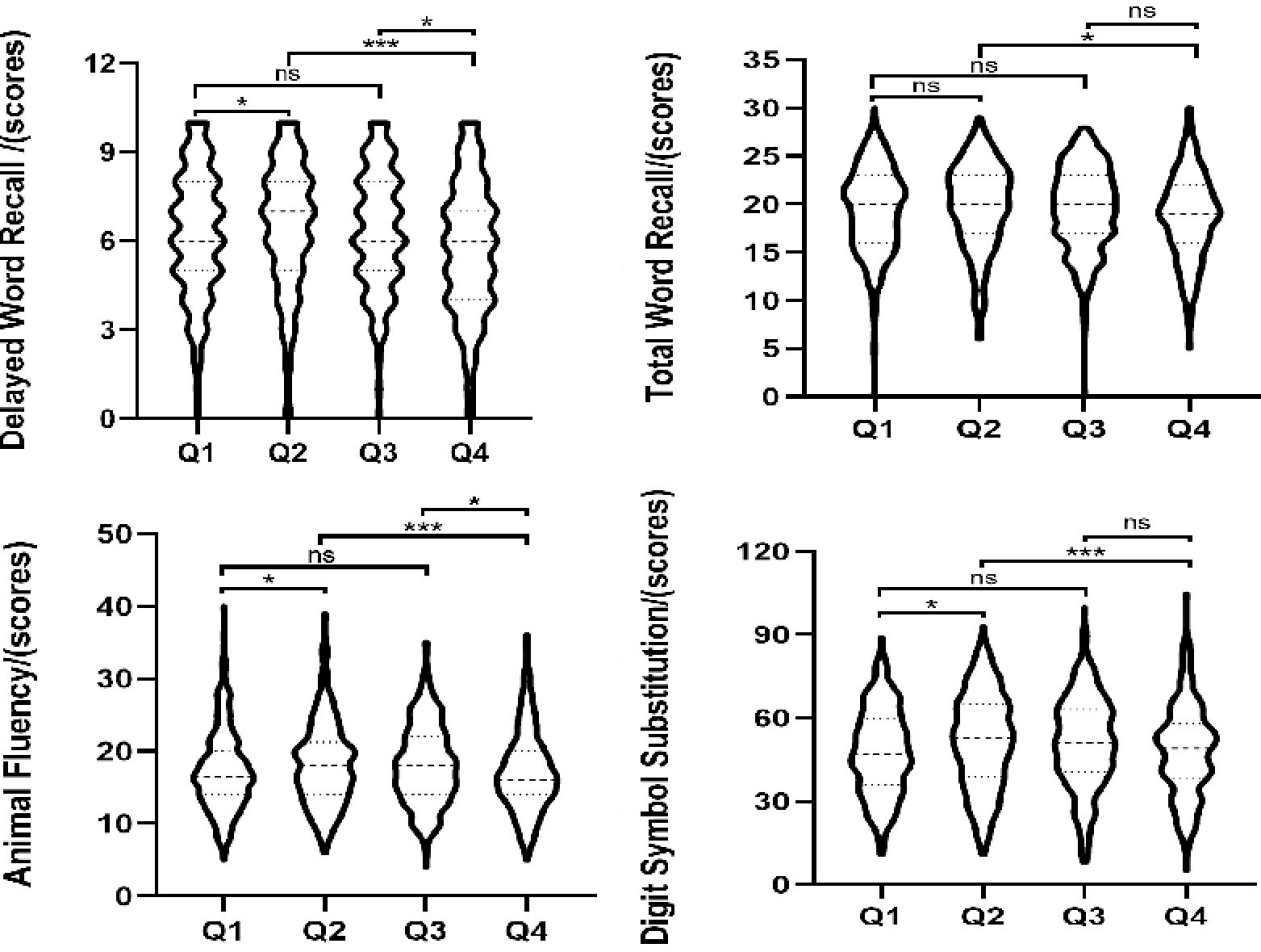
Association between quartiles of SII and cognitive function scores. the first quartile: Q1; the second quartile: Q2; the third quartile: Q3; the fourth quartile:Q4. *P<0.05, ***P<0.001.

As shown in Fig 2, The 3-knot RCS analysis reveal an inverted U-shaped relationship between lgSII levels and cognitive function (*P*_non-liner_<0.05). At a high levels of SII, cognitive function decreases with increasing SII levels. Furthermore, the results shown in Fig 3 show that the quartiles of SII show significant differences in cognitive function (*P* < 0.05). Based on the quartile and RCS results, SII is categorized into two groups: normal group (Q2+Q3) defined as 339.38 < SII ≤ 664.13, and abnormal group (Q1+Q4) defined as SII ≤ 339.38 or SII > 664.13.

### 3.2 Baseline characteristics of the participants

Among 1447 participants included for analysis, 430 (29.72%) individuals had neither abnormal SII nor hyperglycemia, 442 (30.55%) had abnormal SII alone, 73 (5.4%) had prediabetes alone, 198 (13.68%) had diabetes alone, 74 (5.11%) had both abnormal SII and prediabetes and 230 (15.89%) had both abnormal SII and diabetes concurrently. Notably, those who experienced the coexistence of abnormal SII and diabetes tended to be less educated, poorer, more Non-Hispanic Black, higher BMI, lower total energy intake, less Physical activity and nondrinkers. Furthermore, significant differences in the prevalence rates of almost all chronic diseases were observed among these six groups except for age, sex, marital status, smoke, stroke and depression (all *P*<0.05) (Table 1).

### 3.3 Individual cognitive function in the different model

**Fig 4:**
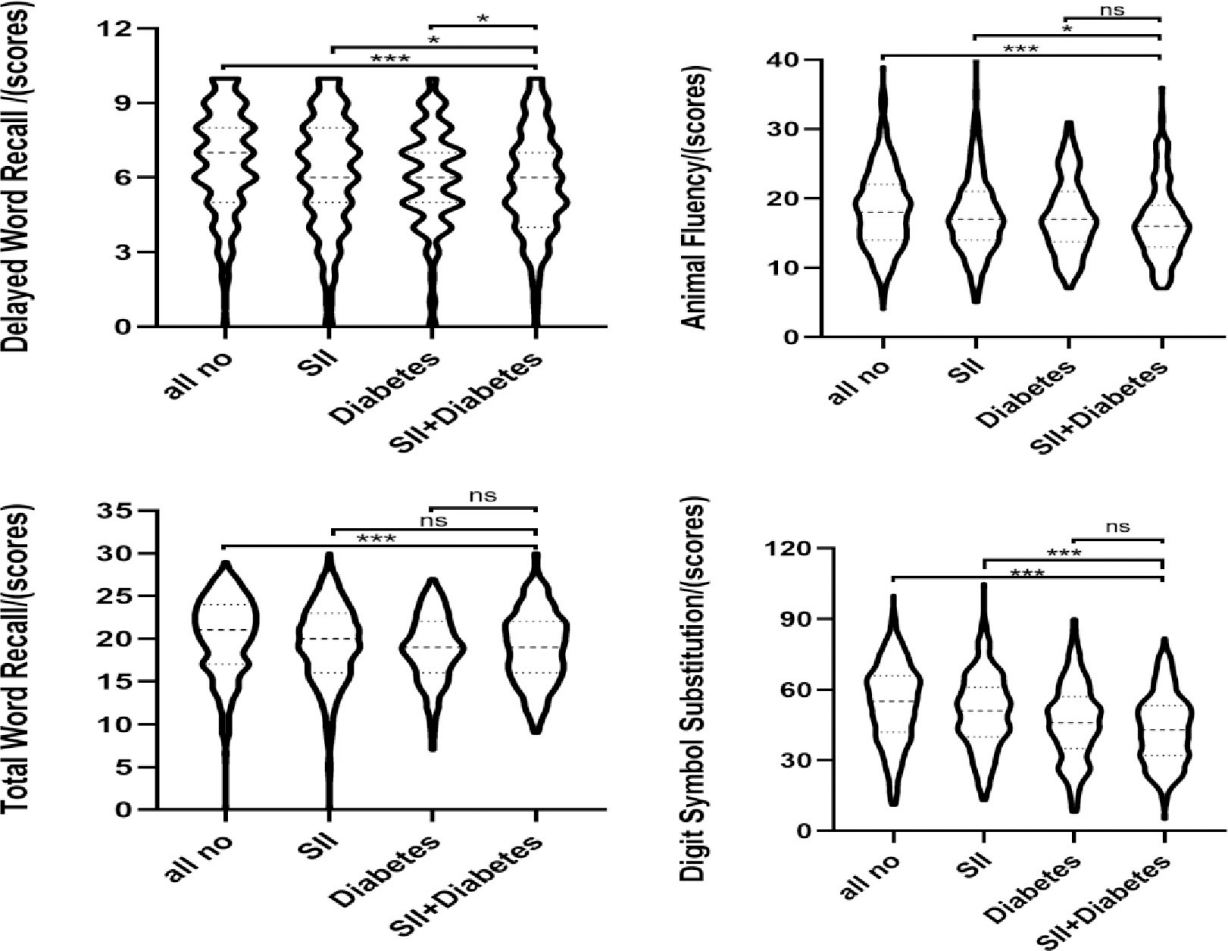
The joint association of abnormal SII levels and diabetes with cognitive function scores. *P<0.05, ***P<0.001.

As illustrated in Fig 4, the four groups exhibited significant differences in various cognitive function tests. However, only in the delayed word recall test was there a significant difference between the Diabetes group and the SII+Diabetes group.

In the unadjusted, model1, model2 and model3, participants in SII+Diabetes group had significantly lower scores in the Delayed Word Recall test compared to the All no group (β: -1.13, -0.76, -0.78 and -0.76). Similar results were obtained in the Digit Symbol Substitution test (β: -11.62, -5.9,5.18 and -5.02). Both unadjusted and model1 analyses revealed that the SII+Diabetes group scored significantly lower than the All no group in Total Word Recall test (β: -1.9 and -0.97), but this association was not significant in model2 and model3. The same results were obtained in the Animal Fluency test (β: -2.78 and -1.15) (Table 2).

**Table 2:** The joint association of abnormal SII levels and pathoglycemia with cognitive function.

	All no	SII	Prediabetes	Diabetes	SII+Prediabetes	SII+Diabetes
**Delayed Word Recall**						
Unadjusted	1(ref)	-0.58(-0.99,-0.17)*	-0.86(-1.52,-0.20)*	-0.63(-1.08,-0.17)*	-0.68(-1.25,-0.12)*	-1.13(-1.58,-0.68)***
Model 1	1(ref)	-0.47(-0.89,-0.06)*	-0.6(-1.37, 0.17)	-0.32(-0.82, 0.18)	-0.44(-0.98, 0.10)	-0.76(-1.18,-0.33)**
Model 2	1(ref)	-0.48(-0.98, 0.02)	-0.59(-1.51, 0.34)	-0.32(-0.95, 0.31)	-0.47(-1.05, 0.11)	-0.78(-1.28,-0.28)*
Model 3	1(ref)	-0.47(-1.11, 0.17)	-0.6(-1.83, 0.62)	-0.31(-1.14, 0.52)	-0.46(-1.22, 0.29)	-0.76(-1.42,-0.10)*
**Total Word Recall**						
Unadjusted	1(ref)	-1.2(-2.04,-0.36)*	-1.81(-3.03,-0.59)*	-1.65(-2.36,-0.93)***	-1.15(-2.54, 0.25)	-1.9(-2.74,-1.07)***
Model 1	1(ref)	-0.98(-1.71,-0.25)*	-1.21(-2.45, 0.03)	-0.88(-1.60,-0.16)*	-0.61(-1.80, 0.58)	-0.97(-1.74,-0.19)*
Model 2	1(ref)	-0.99(-1.87,-0.11)	-1.16(-2.60, 0.29)	-0.72(-1.49, 0.04)	-0.52(-1.87, 0.82)	-0.81(-1.67, 0.05)
Model 3	1(ref)	-0.98(-2.12, 0.15)	-1.26(-3.22, 0.70)	-0.69(-1.70, 0.33)	-0.5(-2.20, 1.20)	-0.72(-1.81, 0.36)
**Animal Fluency**						
Unadjusted	1(ref)	-1.31(-2.15,-0.48)**	-0.7(-2.64, 1.24)	-1.58(-2.77,-0.38)*	-1.77(-3.54,-0.01)	-2.78(-3.77,-1.78)***
Model 1	1(ref)	-1.14(-1.96,-0.33)*	-0.55(-2.34, 1.24)	-0.51(-1.54, 0.51)	-1.34(-3.14, 0.46)	-1.15(-1.92,-0.37)*
Model 2	1(ref)	-1.01(-1.98,-0.03)	-0.25(-2.45, 1.95)	-0.14(-1.27, 0.99)	-1.24(-3.32, 0.85)	-0.82(-1.90, 0.26)
Model 3	1(ref)	-1.01(-2.27, 0.25)	-0.39(-3.18, 2.40)	-0.08(-1.53, 1.38)	-1.25(-3.96, 1.47)	-0.68(-2.15, 0.80)
**Digit Symbol Substitution**						
Unadjusted	1(ref)	-2.91 (-5.88, 0.06)	-5.23(-10.29,-0.16)*	-9.36(-13.36,-5.36)***	-5.65(-10.71,-0.59)*	-11.62(-14.85,-8.39)***
Model 1	1(ref)	-1.94(-4.49, 0.61)	-3.32(-7.87, 1.23)	-5.29(-9.01,-1.57)*	-3.36(-6.82, 0.10)	-5.9(-8.50,-3.29)***
Model 2	1(ref)	-1.64(-4.62, 1.34)	-3.19(-8.31, 1.93)	-4.83(-8.81,-0.86)*	-3.13(-6.91, 0.65)	-5.18(-8.34,-2.03)*
Model 3	1(ref)	-1.63 (-5.51, 2.25)	-3.27(-10.04, 3.51)	-4.75(-10.02, 0.52)	-3.17 (-8.09, 1.75)	-5.02 (-9.21,-0.83)*

AII no is normal SII, non-diabetes and non-prediabetes; SII is abnormal SII, non-diabetes and non-prediabetes; Prediabetes is prediabetes and normal SII; Diabetes is diabetes and normal SII; SII+Prediabetes is abnormal SII and prediabetes; SII+Diabetes is abnormal SII and diabetes.

Values were presented as β(95% confidence interval).

*p<0.05 compared to Group 1

**p<0.01 compared to Group 1

***p<0.001 compared to Group 1

Model 1: Adjusted for age, sex, race, education level, marital status and poverty income ratio.

Model 2: Model 1 + BMI, smoking status, drinking status, hypercholesterolemia, hypertension, coronary heart disease and stroke.

Model3: Model2 + depressive state, Intake of energy and physical activity.

Overall, in the fully adjusted model, both the Diabetes and SII+Diabetes groups exhibited lower cognitive function scores in comparison to the All no group for all tests, with the SII+Diabetes group scoring even lower than the Diabetes group. While both Prediabetes and SII+Prediabetes groups showed decreased cognitive function scores compared to the All no group, there was no significant difference observed (Table 2).

### 3.4 Subgroup analysis

**Fig 5:**
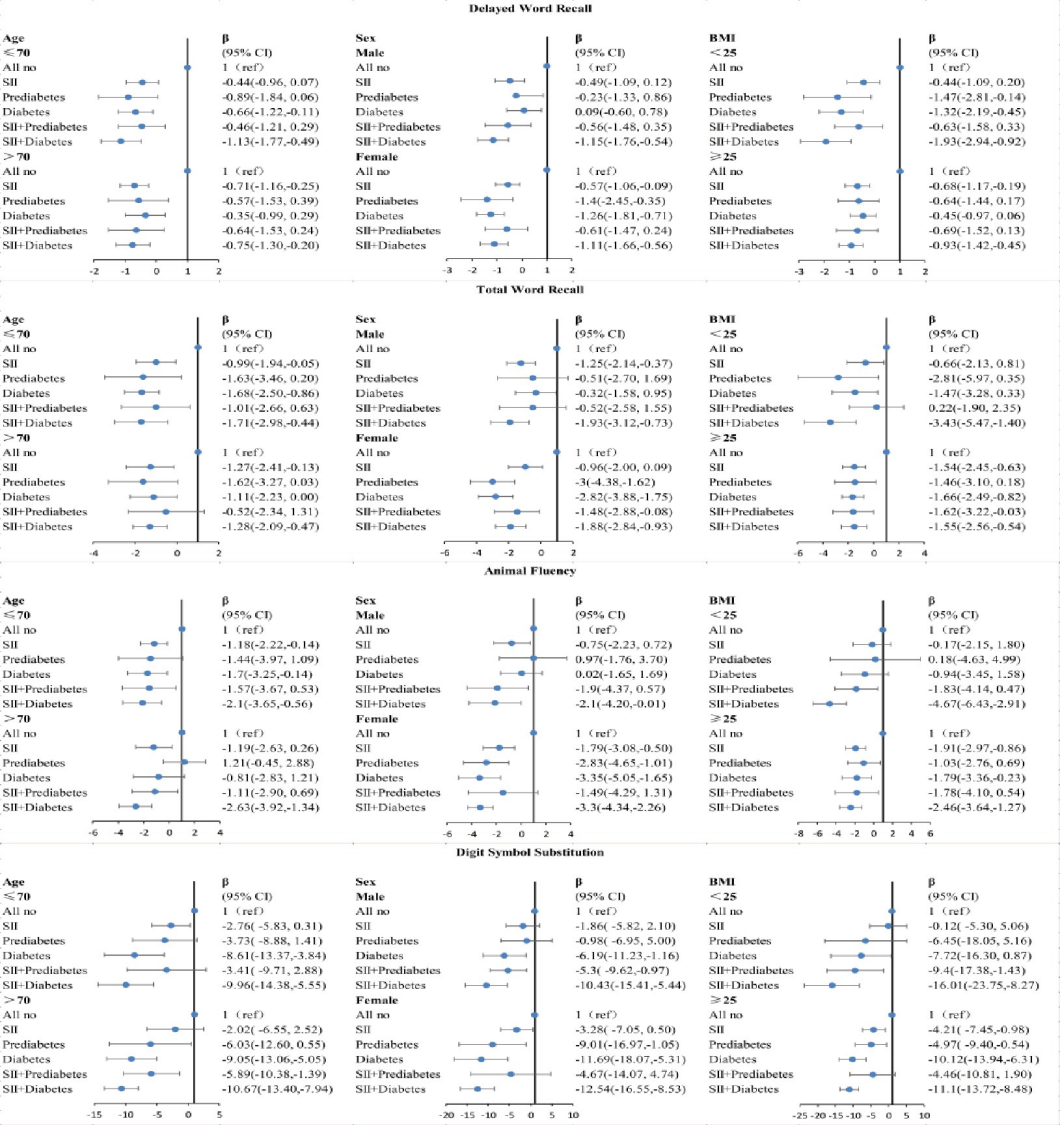
Subgroup analyses of the association of abnormal SII levels and hyperglycemia with cognitive function scores in participants.

The subgroup analyses revealed that participants with concurrent abnormal SII levels and diabetes experienced more pronounced cognitive decline, although the extent of cognitive function reduction varied across different subgroups in different tests (Fig 5). Among all conducted subgroup analyses, participants with both abnormal SII levels and diabetes exhibited the greatest decline in cognitive function compared to those with only diabetes.

In the Delayed Word Recall test and Total Word Recall test, the decrease in cognitive function was more significant among participants ≤70 years old and male. Conversely, in the Animal Fluency test and Digit Symbol Substitution test, the decline in cognitive function was more noteworthy among participants > 70 years old and female. For any test, participants with a BMI <25kg/m^2^ demonstrated a more prominent cognitive decline (Fig 5).

### 3.5 Combined effects of abnormal SII and diabetes on survival rate

**Fig 6:**
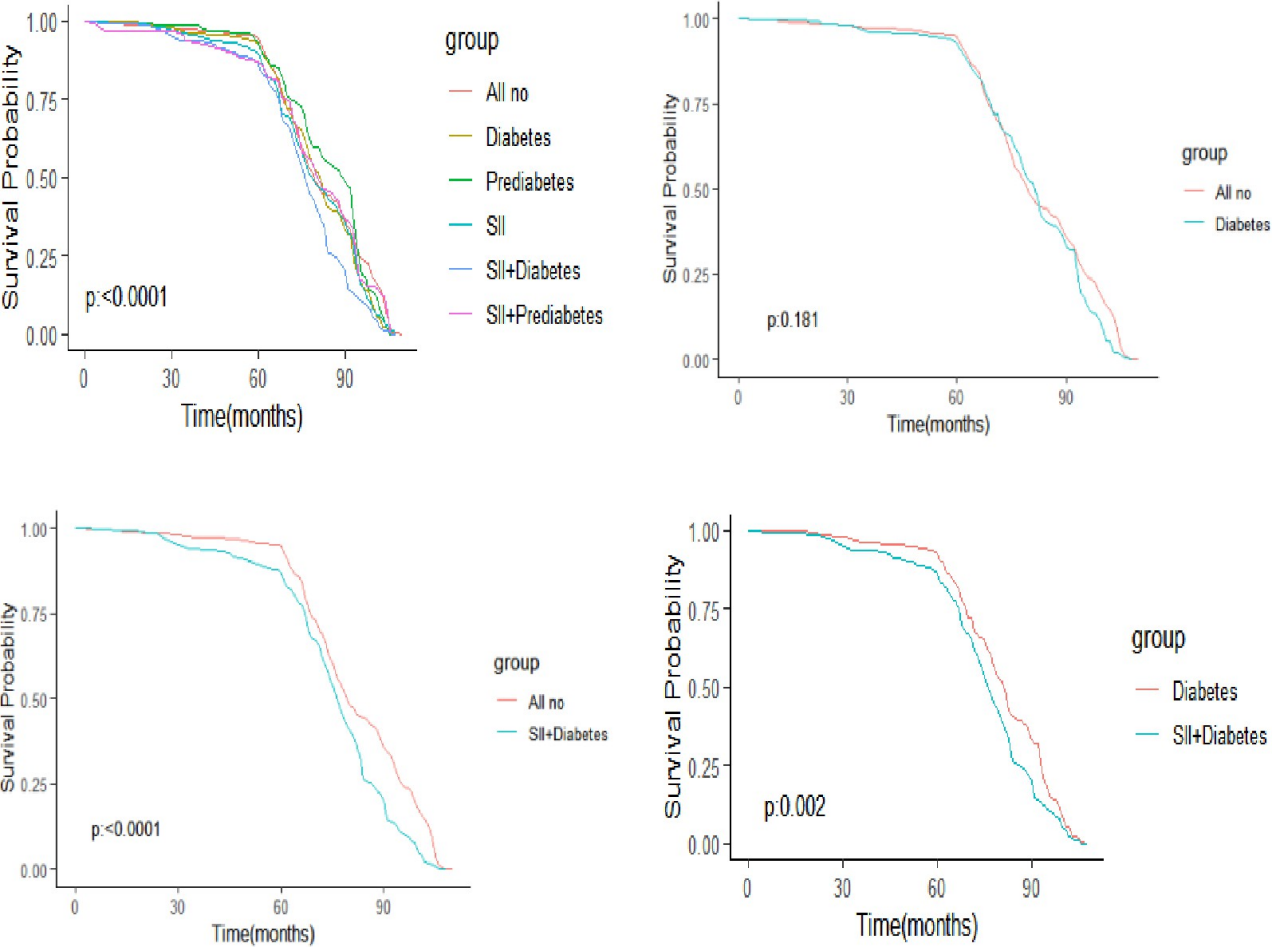
Kaplan–Meier survival curves in six groups in NHANES.

The Kaplan-Meier (KM) plot based on abnormal SII levels and hyperglycemia showed cumulative survival rate for participants (Fig 6). The difference in survival distribution among the six groups was found to be statistically significant (Log-rank test P<0.001); In compared to both the All no group and Diabetes group, the SII+Diabetes group exhibited a significantly lower survival rate. However, diabetes alone did not significantly increase participants’ risk of death.

## 4. Discussion

In this study of older adults in the United States, we observed an inverted U-shaped relationship between lgSII levels and cognitive function. Furthermore, our findings indicate that although abnormal SII and diabetes alone did not significantly contribute to cognitive decline compared to participants with All no group. However, the combined abnormal SII with diabetes (but not prediabetes) did cause a significant impairment in cognitive function compared to All no group. After adjusting for demographic data, health behavior, and past cardiovascular and cerebrovascular events, these results remained significant in the Delayed Word Recall and Digit Symbol Substitution tests. This revealed that the combination of abnormal SII and diabetes may impair delayed memory, executive function and processing speed in elderly Americans. These results were found in Total Word Recall and Animal Fluency tests when adjusting for demographic data, but only showed marginal significance after further adjusting for health behavioral and past cardiovascular and cerebrovascular events. This indicates that when abnormal SII was associated with diabetes, there was no strong correlation with participants’ immediate memory and verbal fluency. What’s more, across all subgroup analyses conducted, individuals with both abnormal SII levels and diabetes exhibited the most significant cognitive decline compared to those affected solely by diabetes. In addition, this study found that abnormal SII levels combined with diabetes further reduced survival rate in older Americans compared to those with normal SII and blood glucose or those diabetes alone.

Numerous studies have shown that chronic inflammation plays a crucial role in the pathogenesis of cognitive impairment in diabetic patients [6,22–24]. Systemic inflammation serves as a crucial mechanism underlying cognitive impairment [25,26]. Cognitive impairment, an important target of vascular damage in diabetes, is associated with both systemic and local brain tissue inflammation involving essential inflammatory cells and molecules. As simple indicators of inflammation, neutrophil to lymphocyte ratio (NLR) [23] and platelet-to-lymphocyte ratio (PLR) [24] have been found to be correlated with the development of cognitive impairment in diabetic patients. Platelets act as an atypical first-line inflammatory biomarker by adhering endothelial cells and leukocytes, thereby modulating the activity of inflammatory components of these cells. Multiple cytokines stem from activated platelets and regulate platelet function in the pathogenesis of cognitive impairment, such as IL-1 and interleukin-6 (IL-6) [25,27,28]. The main components of white blood cells are lymphocytes and neutrophils, which respectively mediate innate and adaptive immunity. Neutrophils, being the majority of white blood cells, play a crucial role in initiating and regulating inflammatory processes. On one hand, activated neutrophils release reactive oxygen species and neutrophilic elastase, which can directly cause damage to brain cells and contribute to the progression of cognitive impairment in diabetic patients [23,28]. On the other hand, activated neutrophils stimulate T lymphocytes by facilitating antigen presentation [23,29]. These T lymphocytes enter the central nervous system through the damaged blood-brain barrier, leading to their numbers within peripheral blood [30]. Besides, T lymphocytes further release tumor necrosis factor α (TNF-α), creating a vicious cycle [23]. The SII is calculated based on three types of circulating immune cells (neutrophils, lymphocytes, and platelets), which are not affected by fluid imbalances [10]. SII provides more valuable information about the relationship between systemic inflammation and cognitive impairment than one or two types of peripheral blood.

SII, which represents the balance of platelets, neutrophils, and lymphocytes, has been recognized as an index of inflammatory status in patients with multifarious chronic inflammatory diseases. Mahemuti N et al. [10] found a significant correlation between SII levels and hyperlipidemia. Topuz MF et al. [31] showed that the SII value was positively correlated with the severity of obstructive sleep apnea patients. Our study demonstrates an inverted U-shaped relationship between lgSII levels and cognitive function. At high levels of SII, cognitive function decreased with increasing SII levels. The reason for low cognitive function at low levels of SII may be attributed to the dual-sided effect of inflammation; excessive activation of neuroinflammation can damage brain cells and neurons, while an appropriate inflammatory state can maintain microenvironment stability [27].

Our study also highlights that diabetes does not increase the risk of cognitive decline in isolation, but rather within a broader spectrum of inflammatory and immune system diseases. Actually, we found that the combination of diabetes and abnormal SII significantly increased the risk of cognitive decline, while neither diabetes nor abnormal SII alone led to a significant risk of this outcome. These results suggest that SII affects cognitive function in a dose-dependent manner. This is similar to the findings of Dove A et al. [32], whose study explained that cognitive impairment is only associated with poorly controlled diabetes (i.e., HbA1c≥7.5%). This indicates that it is the degree of hyperglycemia, rather than diabetes itself, that negatively affects cognitive function. In summary, the effect of diabetes on cognitive impairment is caused by the severity of its complications. Furthermore, our study suggests that inflammation levels may play a role in influencing the impact of diabetes on cognitive function. This is supported by our observation regarding survival rates when considering both diabetes and SII levels together. We found that neither diabetes nor prediabetes status alone significantly reduced participants’ survival rate, only when both diabetes and abnormal SII coexisted did participants’ survival rate show a significantly reduction.

In conclusion, our findings suggest that the combination of abnormal SII levels and diabetes (but not prediabetes) is associated with a greater decline in cognitive function and lower survival rate among older Americans. Implementing interventions targeting these factors may be beneficial in preventing cognitive impairment and mortality. Therefore, further studies are warranted to evaluate the predictive value of abnormal SII levels and diabetes for cognitive impairment and mortality, as well as to conduct prospective studies with extend follow-up periods.
